# Editorial Announcement

**DOI:** 10.3390/nano8010012

**Published:** 2017-12-31

**Authors:** Thomas Nann, Shirley Chiang

**Affiliations:** 1 School of Chemical and Physical Sciences, The MacDiarmid Institute for Advanced Materials and Nanotechnology, Victoria University of Wellington, P.O. Box 600, Wellington 6140, New Zealand; 2 Department of Physics, University of California Davis, One Shields Avenue, Davis, CA 95616-5270, USA

Dear Readers,

After seven exciting years, it is now time for me to step down as founding Editor-in-Chief of *Nanomaterials*. When we started in 2011, the science publication landscape looked very different and I did not anticipate the breakneck speed of change that happened in the following years: the number of publications with the keyword ‘nanomaterial’ almost doubled (according to ‘Web of Science’), open access became mandatory in many countries, established publishing houses adapted their offerings, a multitude of new publishers and journals emerged, and, last but not least, journal metrics became the decisive factor for many authors. At the same time, ‘predatory publishers’ have tried to exploit authors by charging publication fees without providing editorial and publishing services such as peer review, thus chasing authors into the arms of large, established publishers. In other words, the scientific publication market became more competitive and more commercialised.

Given these turbulent circumstances, I am very proud of what the *Nanomaterials* team has achieved. Rigorous quality control and peer review got the budding journal through the storm and the current journal impact factor of 3.5 is a great endorsement for this strategy. Over the last several years, we have observed a consistent increase in the number and quality of submissions and I would also like to take this opportunity to thank our authors for their trust and contribution to the journal. If we extrapolate the current trajectory of the journal, it will see a very bright future indeed.

It is my great pleasure to announce that I will hand over the Editor-in-Chief position of *Nanomaterials* to **Shirley Chiang** of the University of California, Davis, on 31 December 2017. Shirley is an eminent scientist and researcher in the area of scanning probe and electron microscopies. She is interested in the nucleation and growth of metals on surfaces and has an impressive track record in this area of nanomaterials research. I wish Shirley and the *Nanomaterials* team all the best.

With best regards,

Thomas Nann

Dear Readers,

It is a great honour for me to assume the position of Editor-in-Chief of *Nanomaterials*. I congratulate Thomas Nann and the *Nanomaterials* team on the steadily increasing trajectory of the journal, resulting in the current impressive impact factor of 3.553. I look forward to working with the authors, reviewers, and the excellent editorial staff of *Nanomaterials* to continue to improve the journal’s quality and impact.

With best regards,

Shirley Chiang

